# The Efficacy and Safety of Acupuncture for Prophylaxis of Vestibular Migraine: A Study Protocol for a Randomized Controlled Trial

**DOI:** 10.3389/fneur.2021.709803

**Published:** 2021-07-15

**Authors:** Tianye Hu, Hantong Hu, Feng Chen, Bin Jiang, Fengfei Shen, Yingying Su, Mengyi Yang, Jin Hu

**Affiliations:** ^1^Department of Traditional Chinese Medicine and Acupuncture, The First Affiliated Hospital of Jiaxing University, Jiaxing, China; ^2^Department of Acupuncture and Moxibustion, The Third Affiliated Hospital of Zhejiang Chinese Medical University, Hangzhou, China; ^3^Department of Neurology, The First Affiliated Hospital of Jiaxing University, Jiaxing, China

**Keywords:** vestibular migraine, acupuncture, prophylaxis, randomized controlled trial, protocol

## Abstract

**Introduction:** With a high incidence rate and low diagnosis rate, vestibular migraine (VM) can seriously affect the quality of life of patients, but it remains difficult to manage by current treatment options. Acupuncture may be a potential treatment option for VM prophylaxis, but the currently available evidence is still uncertain. Therefore, this trial aims to evaluate the efficacy and safety of acupuncture for VM prophylaxis.

**Methods:** This is a 28-week parallel, randomized, controlled clinical trial including 4 weeks of baseline, 8 weeks of treatment, and 16 weeks of follow-up. A total of 72 participants will be randomly assigned to two groups. The participants will receive acupuncture in the experimental group, while the participants in the control group will be treated with venlafaxine. The primary outcome measures are change in vertigo/migraine days and vertigo/migraine attacks, vertigo severity, and migraine intensity per 4 weeks from baseline. The secondary outcome measures are change in doses of rescue medication, anxiety level, depression level, and quality of life per 4 weeks from baseline. Adverse events will be recorded for safety evaluation.

**Discussion:** This study will investigate the efficacy and safety of acupuncture for VM prophylaxis. The results will contribute to determining whether acupuncture can serve as an optional treatment strategy for treating VM.

**Clinical Trial Registration:** www.ClinicalTrials.gov, identifier: NCT0464088.

## Introduction

Vestibular migraine (VM) is the most common cause of recurrent spontaneous attacks of vertigo, except for benign paroxysmal positional vertigo, which is causally related to migraine (1). Its typical clinical features consist of recurrent episodic vestibular symptoms and migrainous symptoms. Vestibular symptoms can present as vertigo/dizziness, unsteadiness, oscillopsia, and intolerance of head movement, while migrainous symptoms can be characterized by migrainous headache, photophobia, phonophobia, and visual or other neurologic auras (1).

VM can occur at any age, but it is more prevalent in women and in the age group of 30-40 years (2, 3). Epidemiological data of outpatient service show that VM accounts for about 9% of patients in migraine clinics (1), 11% of patients in dizziness clinics (4), and 4.2–29.3% of patients in otolaryngology clinics (5, 6). Despite its high incidence rate, the diagnosed rate of VM is low. As shown in a previous study, referring doctors had suspected only 1.8% of patients to have VM in a tertiary vertigo center, but a diagnosis was finally reached in 20.2% (7). Moreover, VM often leads to some common comorbidities, such as Meniere's disease and psychological disorders (especially anxiety and depression), which contribute to an increasing global burden (8–10). Nevertheless, till now, the pathogenesis of VM has been inconclusive, and further research are urgently warranted. Consequently, there is not any widely acknowledged therapy for treating VM.

At present, the treatment of VM mainly includes rescue treatment and prophylaxis, both of which are mainly pharmacological-based therapies and often lead to a series of unavoidable side effects, such as dizziness, paresthesia, dyspepsia, dry mouth, nausea, somnolence, and chest discomfort (11). Moreover, frequent VM attacks can seriously affect the daily life of patients. Therefore, prophylaxis treatment is very important for VM patients. As a consensus among expert proponents in China, it is vital for VM patients to receive prophylaxis treatment assuming that the frequency of vertigo/migraine episodes is at least three times per month in the past 3 months or the response to rescue therapies is poor in the acute phase of VM (12). Thus, there is a great need to identify alternative preventive treatments for VM.

As one of the major treatment modalities of traditional Chinese medicine (TCM), acupuncture has gained an important role as a non-pharmaceutical therapy for treating a variety of diseases or conditions, thereby receiving increasing worldwide acceptance. Acupuncture may be a potential treatment option for VM prophylaxis, the rationales of which include two major considerations. First, previous studies have reported encouraging results favoring acupuncture for treating vertigo/dizziness (13) and migraine (14, 15), which are major clinical symptoms of VM. Meanwhile, there are several reports indicating that acupuncture is beneficial to psychological disorders such as anxiety and depression (16, 17), which are common comorbidities of VM. Second, emerging studies have revealed that the pathogenesis of VM is related to the dysfunction of the connection between the trigeminal–vestibular–vagus nerves (18, 19). As a form of stimulation, cumulative evidence demonstrates that acupuncture can relieve the symptoms of patients *via* mediating neural plasticity (20). In this scenario, acupuncture can be considered as a potential treatment option for patients with VM, but the current evidence remains inconclusive (21).

In recent years, VM has increasingly attracted the attention of clinicians and researchers; however, there is yet a great lack of randomized controlled trials (RCTs) investigating acupuncture for VM prophylaxis. Therefore, the present study protocol of a RCT aims to investigate the efficacy and safety of acupuncture for VM prophylaxis, which will provide evidence and guide clinicians in this field.

## Method and Analysis

### Study Design

The design of this trial is based on the Standards for Reporting Interventions in Clinical Trials of Acupuncture (22) and the Standard Protocol Items: Recommendations for Interventional Trials (23) reporting guidelines.

**Figure 1:**
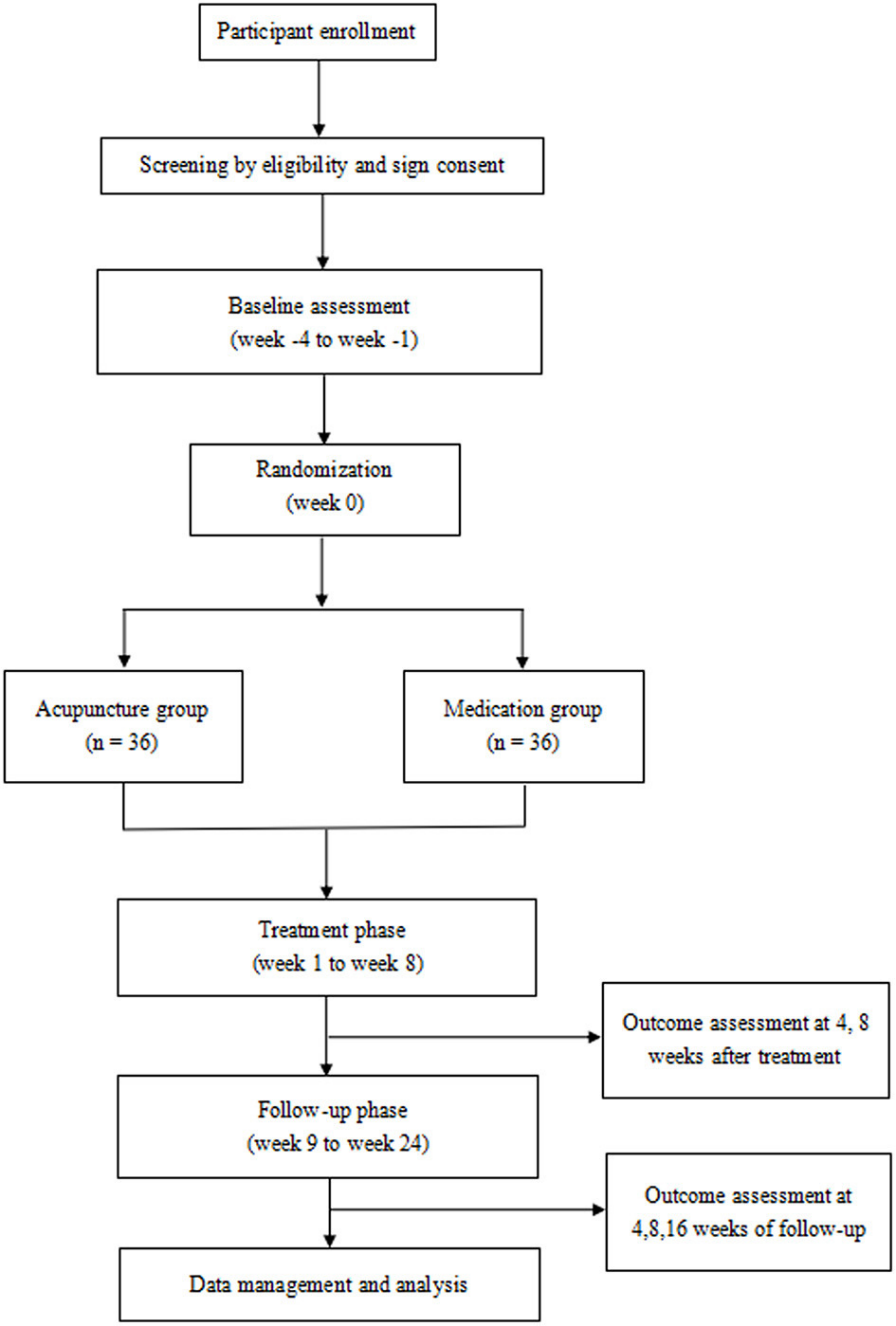
Flow diagram of the study procedure.

This randomized, controlled, single-center clinical trial will comprise two parallel groups. The participants will be randomly assigned in a 1:1 ratio to either the acupuncture group or the control group. The flowchart of the trial is presented in Figure 1, and the schedule of enrolment, interventions, and outcome assessments are presented in Table 1.

**Table 1:** Schedule of enrolment, interventions, and outcome assessments.

**Study period**							
	**Baseline** ** (4 weeks)**	**Allocation**	**Treatment phase** ** (8 weeks)**		**Follow-up phase** ** (16 weeks)**		
Time point	week −4	week 0	Week 4	Week 8	Week 12	Week 16	Week 24
**Enrolment**							
Eligibility screening	×						
Informed consent	×						
Allocation		×					
**Intervention**							
Acupuncture group			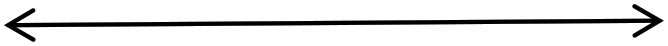				
Medication group			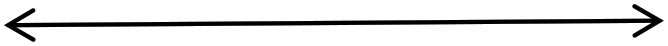				
**Outcome assessments**							
(1) Patient diary (days and numbers of vertigo/migraine)	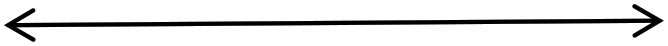		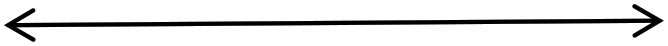		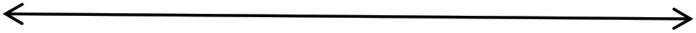		
(2) DHI		×	×	×	×	×	×
(3) VAS		×	×	×	×	×	×
(4) Dosage of rescue medication		×	×	×	×	×	×
(5) GAD-7		×	×	×	×	×	×
(6) PHQ-9		×	×	×	×	×	×
(7) SF-36		×	×	×	×	×	×
Safety evaluation			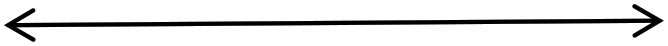		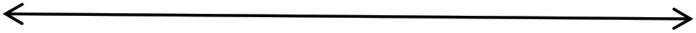		

*×, required; DHI, Dizziness Handicap Inventory; VAS, Visual Analogue Scale; GAD-7, Generalized Anxiety Disorder-7 Scale; PHQ-9, Patient Health Questionnaire Scale; SF-36, 36-Item Short-Form Health Survey*.

### Ethical Standard and Study Registration

This trial is conducted in accordance with the principles of the Declaration of Helsinki and has been approved by the Ethical Committee of The First Affiliated Hospital of Jiaxing University (approval number: LS2020-259). Each participant will be notified regarding the details of the study protocol.

The study protocol has been registered in the clinicaltrials registry, with the identification number NCT0464088.

### Informed Consent

All participants will be given enough time to decide whether to engage in this trial. Patients who participate in this trial will be entitled to the right to achieve all relevant information of the trial, including the benefits or potential risk, and they will be allowed to drop out according to their consent or to discontinue their participation at any point of the study. The confidentiality of all the participant records will be protected. All participants should provide written informed consents if they are included in the trial.

### Study Procedure

The duration of the trial will include three study phases, including a 4-week screening and baseline phase (week −4 to week 0), an 8-week treatment phase (weeks 1-8), and a 16-week follow-up phase (weeks 9-24).

If the participants have an interest to take part in this trial, the researchers will interview their medical history in a screening form. For potentially eligible subjects, they will be instructed to continually keep a patient diary for 4 weeks, which is mainly designed to record details including the number and days of vertigo/migraine attacks, duration, frequency, vertigo severity, migraine intensity, and dose of rescue medication. Subsequently, they will be invited to attend an eligibility assessment to determine whether they can be included in the trial by the study investigators.

### Participant Enrollment

A target sample of 72 participants will be recruited in the acupuncture clinic and neurology clinic at The First Affiliated Hospital of Jiaxing University. The trial will be started from January 2021 to December 2023. Two major strategies will be used to recruit participants with VM. One is to recruit participants in outpatient clinics from the hospital mentioned above. The other is to show recruitment advertisements outside the clinics and through official media platform, which include roll-up banners, WeChat official accounts, and newspapers. The advertisements will contain brief introductions about the inclusion criteria, the population needed to be involved, and the contact information of the researcher.

### Study Population

#### Inclusion Criteria

1. The age is between 18 and 80, and gender is not limited.
2. Patients meet the VM diagnostic criteria proposed by the collaboration of Barany Society and the International Headache Society in 2012 (24).
3. Vertigo/migraine attacks are at least three times per month in the last 3 months or vertigo/migraine days are at least 4 days per month.
4. Patients have an unsatisfactory response to rescue treatments and seek for preventive treatments.
5. Patients can fully understand the study protocol and agree to sign written informed consent forms.

#### Exclusion Criteria

1. The vertigo and headache of patients are caused by other diseases, such as vestibular neuritis, Meniere's disease, tension headache, and other cerebrovascular diseases.
2. Patients have a prophylactic headache treatment with drugs in the past 3 months.
3. Patients are receiving adjunctive therapy that is not widely accepted for treating VM, such as Chinese herbs.
4. Patients have severe complications in cardiovascular, cerebrovascular, liver, kidney, hematopoietic, and other systems that are not controlled significantly.
5. Patients are pregnant and lactating female.
6. Patients have a mental illness that affects cognitive function.

#### Discontinuation or Elimination Criteria

1. Subjects have serious adverse reactions throughout the study and researchers determine it is not safe to continue participating in the trial.
2. Subjects have serious complications or deteriorating conditions throughout the study and emergency measures are urgently needed.
3. Subjects request to withdraw from the trial halfway due to any personal reasons.
4. Subjects have poor compliance and fail to comply with the trial protocol.
5. Subjects receive adjunctive treatments without permission, which will affect the efficacy assessment of current treatment schemes.

### Randomization and Allocation Concealment

Eligible participants will be assigned randomly into the acupuncture group or the control group according to a table of random numbers, which is generated using Statistical Product and Service Solutions software V25.0 (SPSS V25.0).

To guarantee allocation concealment, random sequence will be created and kept secure by a specially designated coordinator who will not participate in other procedures of the trial. The randomization list will only be seen by this specially designated coordinator and will be concealed from other researchers. Grouping information will be hidden using sequentially numbered, opaque, and sealed envelopes. The researchers need to contact the designated coordinator by telephone to obtain the corresponding random number and grouping information for each included participant.

### Blinding

Owing to the complete difference between the two therapies in the two groups (i.e., acupuncture vs. pharmaceutical therapy), the participants will not be blinded in our study. Meanwhile, it is also impractical to blind the acupuncture operators due to the nature of the acupuncture procedure.

Nevertheless, the goal of blinding is to eliminate the influence of subjective factors from study subjects and researchers on the study results. With the attempt to minimize the subjective influence, the treatment, outcome assessment, and statistical analysis will be performed by independent researchers in our study. Statisticians and data managers who collect data and assess outcomes will be blinded to the grouping information by third-party independent personnel.

### Interventions

#### Acupuncture Group

This group will include 36 VM patients. The participants in this group will receive acupuncture treatment for 8 weeks. The participants in the treatment group are not allowed to take prophylactic medications, but in case of intolerable acute VM attacks, the participants in the acupuncture group will be instructed to take triptans as rescue medication, and the dosage of medication will be documented in detail in the medical diary by the patient.

##### Acupoint Selection

The rationales for the acupoint selection are based on consensus with experienced acupuncture experts, which target at alleviating major symptoms (i.e., migraine and vertigo/dizziness) and common comorbidities (i.e., emotional disorders) in VM patients. The acupoint selection of our study is highly overlapping with that in previously published trials involving acupuncture for treating migraine (14, 15), vertigo/dizziness (13, 25), and emotional disorders (16). As shown in Table 2, the selected acupoints include Baihui (DU20), Qianding (DU21), Yintang (EX-HN3), Fengchi (GB20), Shuaigu (GB8), Hegu (LI4), Neiguan (PC6), Shenmen (HT7), Fenglong (ST40), and Taichong (LR3). The location standard of the acupoints is based on the WHO standard for acupuncture point location (26).

**Table 2:** Locations of the selected acupoints for treating vestibular migraine.

**Acupoints**	**Location**
Baihui (DU20)	On the head, 5.0 cun directly above the midpoint of the anterior hairline, or at the midpoint of the line connecting the apexes of the two auricles
Qianding (DU21)	On the head, 3.5 cun directly above the midpoint of the anterior hairline, 1.5 cun anterior to DU 20
Yintang (EX-HN3)	At the forehead, at the midpoint between the two medial ends of the eyebrow
Fengchi (GB20)	On the nape, below the occiput, at the level of DU 16, in the depression between the upper portion of m. sternocleidomastoideus and m. trapezius
Shuaigu (GB8)	On the head, directly above the apex of the auricle and SJ 20, 1.5 cun within the hairline
Hegu (LI4)	On the dorsum of the hand, between the first and second metacarpal bones, in the middle of the second metacarpal bone on the radial side
Neiguan (PC6)	On the palmar aspect of the forearm, 2 cun above the transverse crease of the wrist, on the line connecting PC 3 and PC 7, between the tendons of m. palmaris longus and m. flexor carpi radialis
Shenmen (HT7)	On the wrist, at the ulnar end of the transverse crease of the wrist, in the depression on the radial side of the tendon m. flexor carpi ulnaris
Fenglong (ST40)	On the anterior aspect of the lower leg, 8 cun superior to the external malleolus, lateral to ST 38, two finger-breadth (middle finger) from the anterior crest of the tibia
Taichong (LR3)	On the dorsum of the foot, in the depression proximal to the first metatarsal space

##### Procedures

The acupuncturist will insert sterile, single-use filiform acupuncture needles (length, 40 mm; diameter, 0.30 mm) after disinfecting the skin. The needles used are Hwato needles (Suzhou Hwato Medical Instruments Co., China). Insertion will be followed by the classical needle stimulation methods of lifting/thrusting and twirling/rotating to produce a sensation known as Deqi (a compositional sensation including soreness, numbness, distention, and heaviness). Some acupoints, such as DU20 and DU21, are located beneath the scalp, which could be needled followed by the stimulation methods of pulling and pushing to reinforce a Deqi sensation.

The patients in this group will receive acupuncture once every other day (3 days per week) over an 8-week period (a total of 24 sessions). In each session, the needles will be retained for 30 min.

### Medication Group

This group will include 36 VM patients. The participants in this group will receive an oral administration of venlafaxine at 50 mg once a day for 8 weeks. The reasons for selecting the dose of 50 mg in this study are based on clinical experience and results from a previous study, which revealed that a lower dose (e.g., 37.5 mg) of venlafaxine had a favorable therapeutic effect in the prophylaxis of VM (27). Additionally, a lower dose is likely to have less serious side effects when compared with a higher dose. In case of intolerable acute VM attacks, the participants will be instructed to take triptans as rescue medication, and the dosage of medication will be documented in detail in the medical diary by the patient.

### Outcomes

All outcomes will be assessed at baseline (4 weeks before randomization), at 4 and 8 weeks after intervention, and at 4, 8, and 16 weeks of follow-up.

#### Primary Outcome Measures

1. Change in the number of vertigo/migraine days and vertigo/migraine attacks per 4 weeks from baseline: All participants will be instructed to keep a medical diary to record the number of vertigo/migraine days and vertigo/migraine attacks.
2. Change in vertigo severity per 4 weeks from baseline: Vertigo severity will be measured by the Dizziness Handicap Inventory (DHI). DHI is a well-accepted questionnaire with 25 items and developed to assess the maiming and handicapping impact of dizziness (28). The total DHI index ranges from 0 to 100 (best to worst) to measure the overall severity of vertigo, which include physical, emotional, and functional three dimensions, and the higher the score, the greater effect to the patients.
3. Change in migraine intensity per 4 weeks from baseline: Migraine intensity will be measured by a 10-point Visual Analog Scale (VAS). VAS is the most widely used scale for estimating subjective symptoms, including migraine intensity. The study subjects will use 0–10 (0: no symptoms, 10: most severe symptoms) to rate the intensity of migraine (29, 30).

#### Secondary Outcome Measures

1. Change in doses of rescue medication: Doses of rescue medication (i.e., triptans) will be documented by patients in the medical diary.
2. Change in anxiety level per 4 weeks from baseline: Anxiety level will be measured by Generalized Anxiety Disorder-7 (GAD-7) scale. The GAD-7 is a valid and reliable tool to measure general anxiety symptoms over the past 2 weeks across varied settings and populations (31). A total of seven questions, with frequencies of 0–3, are adopted to assess the presence and severity of anxiety based on the patient's condition, with 0–4 being no GAD, 5–9 being mild GAD, 10–14 being moderate GAD, and 15–21 being severe GAD (32).
3. Change in depression level per 4 weeks from baseline: Depression level will be measured by Patient Health Questionnaire (PHQ-9) scale. The PHQ-9 is generally used to measure depression symptoms over the past 2 weeks with well-established validity and reliability (33). It includes a total of nine questions—the higher the score, the more depressed (34).
4. Change in quality of life per 4 weeks from baseline: Quality of life will be measured by the 36-item Short-Form Health Survey (SF-36). The SF-36 is a tool to evaluate general health-based analysis of life condition in three aspects (functional ability, wellbeing, and overall health) over the past 4 weeks (35). The validity and reliability of SF-36 for measuring quality of life have been proven (36).

### Safety Evaluation

Safety evaluation will be performed based on the number of adverse events (AEs). The participants will report any AE that they experience during the study phase, including acupuncture-related AEs (e.g., discomfort or bruising at the site of needle insertion, vertigo/dizziness, nausea, feeling faint after each treatment, etc.) and drug-related AEs (e.g., dyspepsia, paresthesia, dry mouth, nausea, and somnolence).

### Quality Control

We will take multiple measures to enhance quality control throughout the entire trial process. Prior to the study, we will organize an intensive trial training to ensure that all researchers fully understand the trial protocol, standard operating procedures, and their corresponding roles—for example, all outcome assessors will be trained to perform outcome assessments and fill in the case report forms (CRFs) uniformly. All involved licensed acupuncturists are required to have at least 10 years of clinical experience and will be trained in acupuncture procedure to reduce operation bias as much as possible, especially in positioning of acupoints and acupuncture manipulations.

During the study, we will periodically inspect the implementation of the trial to minimize the occurrence of AEs or complications as much as possible. The reason for dropout or withdrawal will be documented in CRFs. A third-party data safety monitoring committee will be employed to monitor the reliability of the data. They have the right to verify the authenticity and consistency between raw data and recorded data. Meanwhile, all major investigators will organize periodical meetings to discuss issues that will arise during the trial and figure out the best solutions. Moreover, in order to promote patient enrollment, all acupuncture treatment costs will be free for the included participants.

At follow-up visits, economic compensation and health education will be adopted to improve patient compliance and reduce dropouts during a long period of 16-week follow-ups.

### Sample Size Estimation

Power Analysis and Sample Size for Windows (NCSS, Kaysville, Utah, USA; PASS version 15.0) software is used to calculate the required sample size. Owing to the lack of previously published relevant RCTs, the sample size calculation is estimated based on the results of our pilot study. The participants will be divided into two groups in a 1:1 ratio. It is assumed that the mean of the number of vertigo days at 8 weeks after intervention will be 2.2 in the acupuncture group and 3.3 in the control groups, with a pooled standard deviation of 1.54. Given a difference in vertigo days of 1.1 between the two groups and a statistical power of 0.8 to reject a null effect at the 0.05 significance level, the required sample size of each group is 31. Considering an estimated attrition rate of 15%, each group will include 36 initial participants; thereby, a total of 72 participants are needed.

### Statistical Analysis

Statistical software package SPSS19.0 (SPSS Inc., Chicago, IL, USA) will be adopted to conduct the statistical analysis. The statistical analysis will be conducted based on the intention-to-treat principle by third-party statisticians who are blinded to the group allocation and intervention process. Missing data will be imputed by the last-observation-carried-forward method.

Regarding categorical data, these will be displayed as counts and percentages. Numeric data with normal distribution will be expressed as mean ± standard deviations, while data with non-normal distribution will be expressed as median with 95% confidence intervals. For the data in normal distribution, repeated-measures analysis of variance (ANOVA) will be used to perform within-group and between-group comparison by assessing change in continuous variables before and after intervention at different time points, while non-parametric test will be adopted to conduct within-group and between-group comparison for data in on-normal distribution. All hypothesis tests will be bilateral, with *p*-value <0.05 considered statistically significant.

## Discussion

To date, there has not been a universally approved treatment for VM treatment, and VM remains difficult to manage by current treatment options (37). Thus, it is critical to seek an effective and safe non-pharmacological therapy for VM prophylaxis, which can improve the quality of life of VM patients and reduce the side effects of pharmacotherapies and medical expense. On one hand, increasing the trials has favored the therapeutic effect of acupuncture for treating the major symptoms [i.e., vertigo/dizziness (13, 25) and migraine (14, 15)] and common comorbidities [e.g., anxiety (16) and depression (17)] of VM. On the other hand, although the pathogenesis of VM remains inconclusive, it is not only related to the central system but also a kind of recurrent peripheral vestibular disorders (9, 38). Emerging studies (18, 19) have also revealed that VM is related to the dysfunction of the connection between the trigeminal–vestibular–vagus nerves, and cumulative evidence demonstrates that acupuncture can relieve the symptoms of patients *via* mediating neural plasticity (20). In this scenario, acupuncture may be a potential treatment option for VM prophylaxis, but current evidence remains insufficient. Therefore, the present protocol of a RCT aims to investigate the efficacy and safety of acupuncture in the prophylactic treatment of VM patients, which is the first RCT in the acupuncture field.

Given that the acupoint selection has a close correlation with the efficacy maximization of acupuncture, the acupoint prescription of our study is carefully schemed. The rationales for the acupoint prescription are mainly based on consensus with experienced acupuncture experts, which are highly overlapping with those adopted in previously published trials involving acupuncture for treating migraine (15), vertigo/dizziness (13), and emotional disorders (16, 17). The acupoint selection in our study targets at alleviating major symptoms (i.e., migraine and vertigo/dizziness) and common comorbidities (i.e., emotional disorders) in VM patients, given that VM affects both the central and peripheral parts of the nervous system and previous studies revealed that Du meridian plays a vital role in treating various nervous system diseases (39, 40), including vertigo and headache. Accordingly, one important part of our acupoint prescription is to select acupoints of Du meridian [i.e., Baihui (DU20), Qianding (DU21)]. Meanwhile, migraine is one of the major symptoms of VM, and according to the theory of TCM, migraine belongs to *shaoyang* headache, which is corresponding to the gallbladder meridian. Thus, acupoints located in the gallbladder meridian [i.e., Fengchi (GB20), Shuaigu (GB8)] are another important part of our acupoint prescription. Moreover, a great number of experimental and clinical trials reveal the therapeutic effect of acupoints on Du meridian and gallbladder meridian, such as Baihui (DU20) (41) and Fengchi (GB20) (42), for relieving vertigo and migraine, which are two major symptoms in VM patients. In addition, the remaining acupoints [e.g., Yintang (EX-HN3), Neiguan (PC6), Shenmen (HT7), and Taichong (LR3)] are selected to treat clinical symptoms related to psychological disorders, which are common comorbidities in VM patients.

As the controlled arm, venlafaxine is a drug classified as serotonin and norepinephrine reuptake inhibitors, which is widely used to treat depression (43). In recent years, the results of increasing RCTs have favored the efficacy of venlafaxine in the prophylaxis of VM (44). Therefore, we compare the efficacy and safety of acupuncture with venlafaxine, which is chosen as a standard drug therapy in the control group.

It is noteworthy that, owing to two major considerations, sham acupuncture is not selected as the control arm in our study. First, given that acupuncture is very popular and widely used in the hospital that recruited the participants, the overwhelming majority of the study subjects have accumulated a rich experience of acupuncture, which makes successful participant blinding very difficult even if a sham acupuncture group is adopted. Second, despite the fact that sham acupuncture remains to be identified as the method of controlling acupuncture to blind the study subjects and control the non-specific placebo effects, distinguishing between the specific and non-specific effects of acupuncture *via* sham control seems to be impossible. Until now, mimicking acupuncture without any physiological activity is still impractical. Many studies indicate that all currently available modalities of sham acupuncture (regarding penetrating or non-penetrating) are not absolutely inert and have positive treatment effects (45–47). In this case, the overall therapeutic effect of acupuncture may be greatly underestimated if it is compared with sham acupuncture, especially in trials that aim to evaluate the effect of acupuncture for treating pain conditions (48). Therefore, some scholars debate that the complex and multi-element characteristics of acupuncture doom the design of sham acupuncture and that the great effort to control the psychological (non-specific) factors through currently available modalities of sham acupuncture might be unnecessary and unsuccessful (49). In this scenario, we do not set up a sham acupuncture group as the control in our study.

There are several limitations of our study. First, with a lack of objective measurement indicators, till now the diagnostic criteria of VM have been relatively subjective. Meanwhile, its major clinical feature of vertigo is a very subjective symptom; a lot of patients cannot describe it clearly, especially old people, thereby making it difficult to diagnose and judge a therapeutic effect. These factors will have an impact on participant inclusion and outcome assessments. Second, given that there is great lack of previously published relevant RCTs, our sample size is estimated according to our pilot study. Nevertheless, our trial will provide effect size data for sample size calculations of future large-scale RCTs. Third, although sham acupuncture is not adopted as the control, owing to the aforementioned considerations, the psychological (non-specific) effect of acupuncture in treating VM cannot be completely eliminated, owing to the lack of sham acupuncture. Thus, when the trial is finished and the results are interpreted, particular considerations should be given to the psychological (non-specific) effects of acupuncture that may influence the treatment effect.

## Trial Status

The first participant was included in January 2021, and it is expected to complete in December 2023.

## Ethics Statement

The studies involving human participants were reviewed and approved by The First Affiliated Hospital of Jiaxing University. The patients/participants provided their written informed consent to participate in this study. Written informed consent was obtained from the individual(s) for the publication of any potentially identifiable images or data included in this article.

## Author Contributions

TH is responsible for this study. TH and JH designed the trial protocol and drafted the manuscript. HH, FC, and JH revised the manuscript. BJ and FS planned a data analysis solution. YS and MY participated in the recruitment. All authors have read, revised, and approved this version of the manuscript.

## Conflict of Interest

The authors declare that the research was conducted in the absence of any commercial or financial relationships that could be construed as a potential conflict of interest.
